# The Urban–Rural Continuum and Child Health: Influence of Residential Location on Ultra‐Processed Food Consumption, Physical Activity, and Metabolic Biomarkers

**DOI:** 10.1111/phn.70156

**Published:** 2026-07-15

**Authors:** Luiz Felipe da Cruz Rangel, Cynthia Gonçalves Silva, Alessandra Alegre de Matos, Francisco Martins Teixeira, Beatriz Gonçalves Ribeiro

**Affiliations:** ^1^ Laboratory of Research and Innovation in Sports Science and Nutrition Federal University of Rio de Janeiro Macaé Brazil; ^2^ Josué de Castro Institute of NutritionFederal University of Rio de Janeiro Federal University of Rio de Janeiro Rio de Janeiro Macaé Brazil; ^3^ Higher Institutes of Education of CENSA ISECENSA Campos dos Goytacazes Brazil; ^4^ Macaé Campus Federal University of Rio de Janeiro Rio de Janeiro Brazil

**Keywords:** child health, health inequalities, pediatric dyslipidemia, physical activity, urbanization, ultra‐processed foods

## Abstract

**Objective:**

This study aimed to investigate the association between residential location—urban area and urban expansion area and sociodemographic characteristics, health behaviors, and clinical‐metabolic outcomes among schoolchildren aged 6 to 9 years in the municipality of Rio das Ostras, Brazil.

**Methods:**

This was a cross‐sectional study involving 420 students from public municipal schools. Sociodemographic and behavioral data were collected using validated questionnaires (QUADA‐3 and QUAFDA). Anthropometric measurements, blood pressure, and biochemical analyses were conducted. Associations were assessed using Pearson's chi‐square or Fisher's exact tests and logistic regression models, both crude and adjusted for age, sex, maternal and paternal education, and household income.were estimated with 95% confidence intervals (CI).

**Findings:**

Children living in urban expansion areas had lower paternal education levels (*p* = 0.021) and were less likely to engage in active commuting to school (2.4% vs. 42.6%; *p* < 0.001). No significant differences were observed in excess weight, physical activity scores, or ultra‐processed food consumption between the two groups. However, children from urban expansion areas showed a higher prevalence of prediabetes (OR = 0.133; 95% CI = 0.045–0.358; *p* < 0.001), elevated total cholesterol (OR = 0.423; *p* = 0.007), low HDL‐c (OR = 0.324; *p* < 0.001), elevated LDL‐c (OR = 0.304; *p* < 0.001), and elevated triglycerides (OR = 0.484; *p* = 0.029).

**Conclusions:**

Residing in urban expansion areas was associated with worse clinical–metabolic outcomes among schoolchildren, independent of nutritional status, physical activity, and ultra‐processed food consumption. These findings highlight the need for intersectoral and place‐based policies to promote healthier environments for children in territories undergoing urbanization.

## 1 Introduction

The phenomenon of the urban–rural continuum, characterized by the progressive transition and convergence of rural areas toward urbanized contexts, is accompanied by new socioeconomic and environmental dynamics (FAO et al. 2023). Urban expansion areas (UEAs) are transitional territories located between consolidated urban centers and rural zones. Although often overlooked by health surveillance systems, these regions share several characteristics with rural contexts, including limited infrastructure, restricted access to services, and greater social vulnerability. Therefore, understanding child health dynamics in UEAs may provide valuable insights into rural–urban inequalities in low‐ and middle‐income countries (Sen and Gredebäck 2024).

With the advance of urbanization, agri‐food systems have undergone reconfiguration, marked by increased availability, accessibility, and consumption of ultra‐processed foods (Cabrini and Guimarães 2022; Sanmarchi et al. 2023). These products, typically high in calories, fats, sugars, and sodium, and poor in nutritional value, have progressively replaced fresh and minimally processed foods in children's diets (Swinburn et al. 2019). This nutritional transition has been associated with increased prevalence of overweight, dyslipidemia, and insulin resistance, contributing to the early onset of risks related to noncommunicable chronic diseases (Monteiro et al. 2019; Bestari et al. 2023).

In addition to dietary factors, children's physical activity levels may also be negatively affected, especially with regard to school commuting conditions, neighborhood safety, and the availability of structured recreational spaces (Cabral and Cândido 2019; Domogalla et al. 2021). However, understanding these relationships within urbanizing areas remains limited. UEAs are transitional zones where features of consolidated urbanization coexist with infrastructure inherited from rural contexts, posing specific challenges to health promotion.

This scenario is further compounded by structural limitations in public health services in less urbanized regions, such as access barriers, shortages of healthcare professionals, and difficulties in the early detection and follow‐up of clinical and nutritional conditions in children

Given this complexity, it is essential to deepen our understanding of how urbanization influences dietary and physical activity behaviors, as well as clinical–metabolic outcomes in childhood. To our knowledge, this is the first study in Brazil to examine, in an integrated manner, the association between residential location consolidated urban areas (UAs) versus UEAs, ultra‐processed food consumption, physical activity levels, and clinical–metabolic outcomes in schoolchildren. Although grounded in a specific Brazilian context, these findings may reflect global patterns of urbanization and inequalities in child health.

The analysis of these intra‐urban disparities aims to inform the development of more effective public policies that take territorial specificities into account in promoting healthy eating and the early prevention of nutritional and metabolic disorders among child populations. In light of the above, the objective of this study was to examine the association between living in UAs or UEAs and ultra‐processed food consumption, physical activity, and clinical–metabolic outcomes among schoolchildren aged 6 to 9 years residing in the Atlantic Forest region of Rio das Ostras, Brazil.

## 2 Methods

This study employed a cross‐sectional observational design to examine the association between residential location UA versus UEA and socioeconomic factors, consumption of ultra‐processed foods (UPFs), physical activity behaviors, and clinical outcomes such as overweight, obesity, dyslipidemia, elevated blood glucose, and high blood pressure.

The sample comprised 420 schoolchildren aged 6 to 9 years, enrolled in four public schools in the municipality of Rio das Ostras, RJ, selected by the Municipal Department of Education, two located in the UA and two in the UEA. Data collection took place between November and December 2019 and was conducted by a trained and supervised research team.

Inclusion criteria were: enrollment in one of the four selected schools; age between 6 years and 9 years, 11 months, and 29 days; and signed informed consent by parents or legal guardians, along with child assent. Children with physical or intellectual disabilities that prevented participation in the assessments, as well as those taking medications that could interfere with study outcomes, were excluded.

Of the 1205 schoolchildren eligible for the study, 420 signed the informed consent forms and were eligible for assessment. The sample size was calculated using Epi Info software version 7.2 based on the total number of children aged 6 to 10 years (1205). Considering the analysis of multiple outcomes, the sample size was calculated based on an estimated prevalence of 50%, a maximum permissible error of 5%, and a 95% confidence level, resulting in a sample size of 291 children. The total number of children assessed according to anthropometric (*n* = 399), dietary (*n* = 390), and physical activity (*n* = 390) parameters reached a 95% confidence level. Biochemical tests were performed on 223 schoolchildren, representing a 90% confidence level. Socioeconomic questionnaires were administered to 168 guardians, representing an 80% confidence level. The research was interrupted during the COVID‐19 pandemic, with no possibility of returning to schools from 2020 to 2021.

## 3 Socioeconomic Status

Sociodemographic and socioeconomic characteristics were collected at baseline using a standardized questionnaire administered to parents or legal guardians. The variables assessed included maternal self‐reported skin color, maternal and paternal educational attainment (categorized as < 8 years or > 8 years of schooling), and monthly household income (measured in minimum wages: < 1, 1–2, 2–3, or > 3), with the minimum wage at the time of data collection set at R$998.00.

## 4 Ultra‐Processed Food Consumption

Dietary intake was assessed using the third version of the Previous Day Food Questionnaire (QUADA‐3) (Assis et al. 2009). This illustrated single‐day recall questionnaire covers six chronologically ordered daily eating events and includes 21 food items or groups selected for their relevance to school‐aged children. The frequency of consumption of various food types on the previous day was recorded.

Foods and food groups were classified according to the NOVA classification system (Monteiro et al. 2016; Monteiro et al. 2019). The present study focused on the ultra‐processed food (UPF) group, which included products such as chocolate milk, sodas and artificial juices, yogurt, bread and cookies, sweets, packaged snacks, fast food items (e.g., pizza, hamburgers), and French fries. UPF consumption was determined by the number of mentions on the previous day and categorized into tertiles representing low, moderate, and high intake.

## 5 Physical Activity

Physical activity (PA) was assessed using the Previous Day Physical Activity and Food Questionnaire (QUAFDA) (Cabral et al. 2011). The QUAFDA illustrates 11 physical activities (e.g., dancing, walking/running, cycling, climbing stairs, playing ball, jumping rope, swimming, doing gymnastics, skateboarding, helping with household chores, and playing with a dog) across three different intensity levels (slow, fast, and very fast). A PA score was generated by assigning weights of one, three, and nine to reported activities, representing light, moderate, and vigorous intensity, respectively. As there is no standardized cutoff, the overall PA level was categorized based on tertiles of the score distribution (least active, intermediate, and most active).

The QUAFDA also assessed the mode of commuting to school, with five response options grouped into active commuting (walking and cycling) and passive commuting (bus, car, and motorcycle). For the analyses, active commuting was classified as yes or no.

## 6 Clinical Variables

Weight and height were measured by trained personnel using a portable stadiometer (Altura Exata) and a portable digital scale (Tanita). Body mass index (BMI) was calculated as weight in kilograms divided by height in meters squared (kg/m^2^), and children were classified into weight categories (underweight, normal weight, overweight, and obesity) using *z*‐scores, according to the World Health Organization (WHO) criteria (Onis et al. 2007).

Blood pressure (BP) was measured using a validated digital device (OMRON HEM‐705 CP) with appropriately sized cuffs, following the guidelines of the Brazilian Society of Cardiology (Sociedade Brasileira de Cardiologia 2016). Two measurements were taken with a 2 min interval, and the mean value was used for classification. High BP was defined as values at or above the 95th percentile for age, sex, and height percentile.

Venous blood samples were collected after a 12 h fast. Samples were centrifuged, stored in thermal boxes, and transported to the laboratory within 2 h, where they were frozen for later analysis. The lipid profile including triglycerides (TG), total cholesterol (TC), low‐density lipoprotein cholesterol (LDL‐c), and high‐density lipoprotein cholesterol (HDL‐c) and fasting glucose were analyzed using the enzymatic colorimetric method with the LABEST kit. LDL‐c was calculated using the Friedewald formula. Classification cutoffs followed the guidelines of the Brazilian Society of Cardiology (TC > 170 mg/dL, LDL‐c > 110 mg/dL, HDL‐c < 45 mg/dL, and TG > 75 mg/dL) (Précoma et al. 2019) and the Brazilian Diabetes Society (normoglycemia < 100 mg/dL; prediabetes ≥ 100 and < 126 mg/dL; diabetes ≥ 126 mg/dL) (Sociedade Brasileira de Diabetes 2021).

## 7 Statistical Analysis

All variables were subjected to descriptive analysis, with absolute and relative frequencies calculated for categorical variables, and means and standard deviations for continuous variables. UPF consumption was expressed as a total score for the previous day and categorized into tertiles. The final PA score was calculated by summing the scores of the 11 activities and also categorized into tertiles. To compare sociodemographic factors, UPF consumption, PA levels, and commuting type between residential locations (UA and UEA), the chi‐square test of independence and Fisher's exact test were used.

The association between residential location (UA vs. UEA) and clinical outcomes (overweight, fasting glucose, lipid profile, and BP) was assessed using logistic regression models. Crude and adjusted odds ratios (OR) with 95% confidence intervals (95% CI) were estimated. For the adjusted models, the covariates age, sex, maternal education, paternal education, and household income (in minimum wages) were included. Binary logistic regression was used for dichotomous outcomes (overweight, dyslipidemia, elevated glucose, and high BP), while multinomial logistic regression was applied to outcomes with more than two categories. All analyses were conducted using R software, version 4.3.0, with a significance level of 5%.

## 8 Ethical Considerations

The study was approved by the Research Ethics Committee of the Federal University of Rio de Janeiro—Macaé campus, under protocol number 3.706.212, in accordance with the guidelines of the National Health Council of the Brazilian Ministry of Health. All parents or guardians received their children's evaluation results and were advised to seek professional follow‐up in cases of clinical abnormalities.

## 9 Results

Table 1 shows that a total of 420 schoolchildren were assessed, with a mean age of 7.9 years. The majority resided in urban areas (UA) (78.3%), while 21.7% lived in urban expansion areas (UEA). The sample included 220 girls (52.38%) and 200 boys (47.6%).

**TABLE 1:** Sociodemographic, socioeconomic, lifestyle, and clinical conditions in schoolchildren aged 6 to 9 years, Rio das Ostras, RJ, Brazil. (*n* = 420).

Variable	*n* (%)
**Location (*n* = 420)**	
Urban expansion area (UEA)	91 (21.67)
Urban area (UA)	329 (78.33)
**Sex (*n* = 420)**	
Female	220 (52.38)
Male	200 (47.62)
Age (*n* = 420)	Mean
UEA	7.67
UA	7.97
**Mother's race/Ethnicity (*n* = 167)**	
Mixed‐race	100 (59.88)
White	41 (24.55)
Black	23 (13.77)
Yellow	2 (1.20)
Indigenous	1 (0.60)
**Mother's Education (*n* = 162)**	
More than 8 years	109 (67.28)
Less than 8 years	53 (32.72)
**Father's Education (*n* = 159)**	
More than 8 years	80 (50.31)
Less than 8 years	76 (47.80)
No formal education	3 (1.89)
**Income (in minimum wages) (*n* = 157)** ^#^	
More than 1 up to 2 wages	68 (43.31)
Zero up to 1 wage	62 (39.49)
More than 2 up to 5 wages	27 (17.20)
**Active commuting to school (*n* = 387)**	
No	255 (65.90)
Yes	132 (34.10)
**Consumption of UPFs c (*n* = 387)**	
No	5 (1.30)
Yes	382 (98.70)
**UPFs c consumption (tertiles) (*n* = 382)**	
Low consumption	143 (37.40)
Moderate consumption	117 (30.60)
High consumption	122 (31.90)
**Engaged in physical activity (*n* = 381)**	
No	10 (4.13)
Yes	371 (95.87)
**Physical activity—Tertile (371)**	
Less active	124 (33.40)
Moderate	127 (34.20)
More active	120 (32.40)
**BMI‐for‐age classification d (*n* = 399)**	
Underweight/Normal weight	279 (69.92)
Overweight	61 (15.29)
Obesity/Severe obesity	59 (14.79)
**Blood Pressure (*n* = 377)**	
Normal	279 (74.01)
Elevated/Hypertension	98 (25.99)
**Fasting glucose (*n* = 224)**	
Normal	205 (91.52)
Prediabetes	19 (8.48)
**Total cholesterol (TC) e (*n* = 223)**	
Acceptable	133 (59.64)
Elevated	90 (40.36)
HDL‐c f (*n* = 223)	
Acceptable	128 (57.40)
Low	95 (42.60)
**LDL‐c g (*n* = 223)**	
Acceptable	147 (65.92)
Elevated	76 (34.08)
**Triglycerides (TG) (*n* = 223)**	
Acceptable	96 (43.05)
Elevated	127 (56.95)

^a^ UEA, Urban Expansion Area; ^b^UA, Urban Area; ^c^UPFs, Ultra‐processed foods; ^d^BMI‐for‐age, Body Mass Index by age; ^e^TC, Total cholesterol; ^f^HDL‐c, High‐density lipoprotein cholesterol; ^g^LDL‐c, Low‐density lipoprotein cholesterol; ^#^, Official minimum wage during the study period (2019) = R$998.00.

Among mothers, the most commonly self‐reported skin color was *parda* (mixed race) (59.9%), followed by white (24.6%) and Black (13.8%). Maternal education of more than eight years was more prevalent (67.3%) than paternal education (50.3%), with 1.9% of fathers having no formal education.

Regarding household income, most families lived on up to two minimum wages per month, with 43.3% reporting income between one and two minimum wages and 39.5% reporting income of up to one minimum wage. Only 17.2% had an income above two minimum wages.

In terms of health behaviors, most children used passive transportation to school (65.9%), ultra‐processed food (UPF) consumption was nearly universal (98.7%), and 95.9% reported engaging in some form of PA.

Regarding clinical profiles, 30.1% of children were overweight, with 15.3% classified as overweight and 14.8% as obese or severely obese. Additionally, 26% had elevated BP or hypertension, and 8.5% had fasting blood glucose levels consistent with prediabetes. TC was elevated in 40.4% of the children, HDL‐c was below recommended levels in 42.6%, and LDL‐c was elevated in 34.1%. Finally, more than half of the children (57.0%) had elevated triglyceride levels.

The analysis of sociodemographic and socioeconomic characteristics of the schoolchildren by residential location (Table 2) revealed no significant differences regarding sex, maternal self‐reported race/skin color, household income, or maternal education. On the other hand, a significant association was observed between residential location and paternal education (*p* = 0.021; effect size = 0.212).

**TABLE 2:** Association between place of residence and sociodemographic and socioeconomic factors among schoolchildren aged 6 to 9 years, Rio das Ostras, RJ, Brazil.

Variable	UEA^a^ (*n* = 91)	UA^b^ (*n* = 329)	*p*‐value
**Sex—*n* (%)**			
Male	47 (51.65)	153 (46.50)	0.453^c^
Female	44 (48.35)	176 (53.50)	
**Mother's race/Ethnicity—*n* (%)**			
White	17 (23.94)	24 (25.00)	0.603^d^
Mixed‐race	42 (59.15)	58 (60.42)	
Black	10 (14.08)	13 (13.54)	
Yellow	2 (2.82)	0 (0.00)	
Indigenous	0 (0.00)	1 (1.04)	
**Mother's Education—*n* (%)**			
More than 8 years	45 (66.18)	64 (68.09)	0.932^c^
Less than 8 years	23 (33.82)	30 (31.91)	
**Father's Education—*n* (%)**			
More than 8 years	27 (40.91)^*^	53 (56.99)^*^	0.021^d^
Less than 8 years	39 (59.09)^*^	37 (39.78)^*^	
**Income (in minimum wages)—*n* (%)** ^#^			
Zero to 1 wage	24 (38.10)	38 (40.43)	0.384^c^
More than 1 up to 2 wages	25 (39.68)	43 (45.74)	
More than 2 up to 5 wages	14 (22.22)	13 (13.83)	

^a^ UEA, Urban Expansion Area;^b^UA, Urban Area; ^#^Official minimum wage during the study period (2019) = R$998.00. ^c^Chi‐square independence test. ^d^Fisher's exact test. ^*^Indicates statistically significant differences between localities (*p* < 0.05).

With regard to behaviors reported for the previous day, no significant associations were found when analyzing UPF consumption patterns or PA levels among schoolchildren from different residential areas. In contrast, a strong association was observed between residential location and active commuting to school (*p* < 0.001). Nearly all children living in the UEA used passive commuting (97.6%), whereas this proportion was 57.4% among those residing in the UA (Table 3).

**TABLE 3:** Association between place of residence and behaviors related to UPF consumption, physical activity, and school commuting among schoolchildren aged 6 to 9 years, Rio das Ostras, RJ, Brazil (*n* = 420).

Variable	AEU^a^	AU^b^	*p*‐value
**UPF^c^ Consumption**			0.1801^c^
Low consumer	25 (31.6%)	118 (38.9%)	
Moderate consumer	22 (27.8%)	95 (31.4%)	
High consumer	32 (40.5%)	90 (29.7%)	
**Physical activity—score**			0.1161^c^
1st tercile—Least active	33 (42.3%)	91 (31.1%)	
2nd tercile—Intermediate	26 (33.3%)	101 (34.5%)	
3rd tercile—Most active	19 (24.4%)	101 (34.5%)	
**Active commuting to school**			0.0001^c^
No	80 (97.6%)	175 (57.4%)	
Yes	2 (2.4%)	130 (42.6%)^*^	

^a^ UEA, Urban Expansion Area; ^b^UA, Urban Area; ^c^UPFs, Ultra‐processed foods; ^c^Chi‐square independence test; ^*^Indicates statistical differences between localities. *p*‐value < 0.05.

Regarding clinical analyses, no significant associations were found between residential location and nutritional status based on BMI‐for‐age or BP. In contrast, altered fasting glucose (prediabetes) was significantly more frequent among schoolchildren living in the UEA, with children in the UA showing an 87% lower odds of presenting this condition (OR = 0.133; 95% CI = 0.045–0.358; *p* < 0.001). Similar results were observed for lipid parameters. The odds of having elevated TC were significantly lower among children from the UA (OR = 0.423; 95% CI = 0.225–0.786; *p* = 0.007). Likewise, the odds of having low HDL‐c were significantly lower in the UA (OR = 0.324; 95% CI = 0.170–0.606; *p* < 0.001), as were the odds of elevated LDL‐c (OR = 0.304; 95% CI = 0.160–0.573; *p* < 0.001). Regarding triglycerides, children residing in the UA had a significantly lower risk of elevated levels, with an OR of 0.484 (95% CI = 0.247–0.916; *p* = 0.029) (Table 4).

**TABLE 4:** Odds Ratio (OR) for clinical outcomes associated with place of residence among schoolchildren aged 6 to 9 years in Rio das Ostras, RJ, Brazil (*n* = 420).

Dependent Variable	AEU^a^ (*n* = 91)	AU^b^ (*n* = 329)	OR^**^	95% CI^***^	*p*‐value
**BMI/Age Classification** ^d^					
Thinness/Normal weight	54 (70.1%)	202 (68.9%)	—	—	—
Overweight	12 (15.6%)	46 (15.7%)	1.025	0.508; 2.069	0.945
Obesity	11 (14.3%)	45 (15.4%)	1.094	0.530; 2.257	0.808
**Blood pressure**					
Normal	55 (68.8%)	224 (75.4%)	—	—	—
Elevated/Hypertension	25 (31.2%)	73 (24.6%)	0.717	0.420; 1.246	0.229
**Fasting glucose**					
Normoglycemia	43 (76.8%)	149 (96.1%)	—	—	—
Pre‐diabetes	13 (23.2%)	6 (3.9%)	0.133	0.045; 0.358	< 0.001
**Total cholesterol (TC)** ^e^					
Acceptable	25 (44.6%)	101 (65.6%)	—	—	—
Elevated	31 (55.4%)	53 (34.4%)	0.423	0.225; 0.786	0.007
**HDL‐c** ^f^					
Acceptable	21 (37.5%)	100 (64.9%)	—	—	—
Low	35 (62.5%)	54 (35.1%)	0.324	0.170; 0.606	< 0.001
**LDL‐c** ^g^					
Acceptable	26 (46.4%)	114 (74.0%)	—	—	—
Elevated	30 (53.6%)	40 (26.0%)	0.304	0.160; 0.573	< 0.001
**Triglycerides (TG)**					
Acceptable	17 (30.4%)	73 (47.4%)	—	—	—
Elevated	39 (69.6%)	81 (52.6%)	0.484	0.247; 0.916	0.029

^a^ UEA, Urban Expansion Area; ^b^UA, Urban Area; ^d^BMI‐for‐age, Body Mass Index by age; ^e^TC, Total cholesterol; ^f^HDL‐c, High‐density lipoprotein cholesterol; ^g^LDL‐c, Low‐density lipoprotein cholesterold ^**^OR, Odds Ratio; ^***^CI, Confidence Interval. * Reference category for the odds ratio: Urban Area (AUb). Binary logistic regression models. *N* = 420. *p*‐value < 0.05.

After adjustment for age, sex, maternal education, paternal education, and household income, the associations between residing in UEA and metabolic alterations (elevated glucose, TC, HDL‐c, and LDL‐c) remained significant, indicating lower odds for schoolchildren living in urban areas. No significant associations were observed for overweight and elevated BP. In the case of elevated triglycerides, a borderline association was identified (*p* = 0.068) (Table 5).

**TABLE 5:** Adjusted Odds Ratio (OR) for clinical outcomes associated with place of residence among schoolchildren aged 6 to 9 years in Rio das Ostras, RJ, Brazil (*n* = 420).

Dependent variable	AEU^a^	UA^b^	Adjusted OR^**^	95% CI^***^>	*p*‐value
**BMI/Age classification** ^d^					
Thinness/Normal weight	54 (70.1%)	202 (68.9%)	—	—	
Overweight/Obesity	23 (29,9%)	91 (31,1%)	1.293	0.563–2.967	*p* = 0.544
**Blood pressure**					
Normal	55 (68.8%)	224 (75.4%)	—	—	
Elevated/Hypertension	25 (31.2%)	73 (24.6%)	0.701	0.314–1.563	*p* = 0.385
**Fasting Glucose**					
Normoglycemia	43 (76.8%)	149 (96.1%)	—	—	
Pre‐diabetes/Diabetes	13 (23.2%)	6 (3.9%)	0.171	0.047–0.623	*p* = 0.007
**Total Cholesterol (TC)** ^e^					
Acceptable	25 (44.6%)	101 (65.6%)	—	—	
Elevated	31 (55.4%)	53 (34.4%)	0.366	0.161–0.831	*p* = 0.016
**HDL‐c** ^f^					
Acceptable	21 (37.5%)	100 (64.9%)	—	—	
Low	35 (62.5%)	54 (35.1%)	0.166	0.069–0.399	*P* < 0.001
**LDL‐c** ^g^					
Acceptable	26 (46.4%)	114 (74.0%)	—	—	
Elevated	30 (53.6%)	40 (26.0%)	0.243	0.102–0.578	*p* = 0.001
**Triglycerides (TG)**					
Acceptable	17 (30.4%)	73 (47.4%)	—	—	
Elevated	39 (69.6%)	81 (52.6%)	0.469	0.208–1.059	*p* = 0.068

^a^UEA, Urban Expansion Area; ^b^UA, Urban Area; ^d^BMI‐for‐age, Body Mass Index by age; ^e^TC, Total cholesterol; ^f^HDL‐c, High‐density lipoprotein cholesterol; ^g^LDL‐c, Low‐density lipoprotein cholesterol. ^**^OR, Odds Ratio; ^***^CI, Confidence Interval. ^*^Reference category for the odds ratio: Urban Area (UA^b^). Binary logistic regression models adjusted for age, sex, maternal and paternal education, and household income. *N* = 420. *p*‐value < 0.05.

## 10 Discussion

The present study identified a significant association between residence in urban expansion areas (UEAs) and lower paternal educational attainment, highlighting a less visible dimension of social inequality that affects the family environment of children. Although maternal education and household income did not differ significantly between areas, the lower educational level of fathers in UEAs may reflect a specific pattern of educational vulnerability, not necessarily accompanied by other economic deprivations. This finding is relevant, as paternal education has been linked to reduced availability of time, lower academic support, and less encouragement of healthy practices at home—all of which can negatively impact children's development and health (Woźniak et al. 2022; Sarwar et al. 2024).

The near‐universal consumption of UPFs among schoolchildren reflects a structural dietary shift that transcends socioeconomic boundaries. The presence of such products in children's diets points to a phenomenon characterized by wide availability, economic accessibility, and aggressive marketing, particularly targeting children. This finding is consistent with national and international data highlighting the alarming rise in childhood UPF consumption. In Brazil, the 2018–2019 Household Budget Survey reported that 19.7% of total caloric intake among individuals aged ten and older came from these foods (Instituto Brasileiro de Geografia e Estatística [IBGE] 2020). Studies from Nigeria, Mexico, and the United Kingdom (Oguizu and Mbakwe 2023; Kamanga et al. 2022; Chang et al. 2021) reinforce this trend in different global contexts.

In UEAs, this phenomenon is even more concerning, as it indicates the loss of protective eating behaviors historically associated with rural environments, such as greater consumption of fresh foods and home‐prepared meals. Given the association between UPF intake and metabolic outcomes, such as dyslipidemia and glycemic alterations (Petridi et al. 2024), there is a clear need for universal regulatory public policies, including restrictions on child‐targeted advertising, improved nutritional labeling, and the strengthening of school feeding programs as platforms for promoting healthy eating habits.

No significant association was observed between residential location and overall PA levels. However, children in UEAs were more frequently in the lowest PA tertile, suggesting a tendency toward reduced engagement in vigorous activities. This apparent similarity in overall PA masks a stark contrast in commuting patterns: the sharp decline in active commuting among children in UEAs underscores the impact of infrastructural barriers that constrain daily mobility. This contrast highlights the reduction of active mobility behaviors in UEAs, without the compensatory infrastructure available in consolidated urban settings (Llamas et al. 2023; Barrés et al. 2022).

Similar findings have been reported in international studies. In Canada, for example, only 5% of rural children used active commuting, compared to 40% in urban zones (Vitale et al. 2019). Conversely, studies in Portugal (Rodrigues et al. 2018) and Mexico (Hernández et al. 2019) reported different patterns, with higher prevalence of active commuting even outside urban centers—suggesting that the relationship between territory and active mobility is shaped by contextual variables such as public policy, local culture, and infrastructure availability. In the present study, the influence of the municipal policy providing free school transportation in UEAs is also notable. While essential for ensuring access to education, it may further reduce opportunities for active commuting among children in these areas. These findings reinforce the need for territorialized interventions that address the environmental and social barriers to PA in urban expansion zones.

No significant differences were observed between UAs and UEAs in the prevalence of excess weight (30%) and obesity (15%), values that already reflect the high burden of these outcomes among Brazilian children (Instituto Brasileiro de Geografia e Estatística [IBGE] 2010). Although not differing between areas, the high prevalence of elevated BP highlights that cardiometabolic risks are already widespread in childhood, adding to the concerning scenario observed in UEAs. However, the analysis of metabolic markers revealed a particularly relevant result: children living in UAs were less likely to present multiple clinical alterations such as prediabetes, dyslipidemias, and hypertriglyceridemia compared with their peers from UEAs. What distinguishes UEAs, therefore, is the additional burden of metabolic alterations, suggesting that schoolchildren in these transitional areas accumulate cardiometabolic risks beyond those expected from excess weight alone.

The associations observed for elevated glucose, TC, HDL‐c, and LDL‐c remained significant after adjustment for age, sex, maternal education, paternal education, and household income, indicating the robustness of the findings. The magnitudes of the odds ratios did not differ substantially between crude and adjusted models, reinforcing that the effect of residential location on metabolic outcomes is not explained solely by sociodemographic and socioeconomic factors.

These results indicate greater metabolic vulnerability among schoolchildren in UEAs, even in the absence of differences in nutritional status, dietary intake, or PA levels—suggesting that the accumulation of cardiometabolic risks may occur silently and early in these territories. The prevalence of dyslipidemia in this sample was high (83%), consistent with Brazilian studies reporting rates between 50% and 88% in different regions (Teixeira et al. 2017; Quadros et al. 2016; Vergara et al. 2019; Maia et al. 2020; Gomes et al. 2020). In line with these findings, a study in Wuhan, China, reported higher glucose and triglyceride levels among rural boys (Mccarthy et al. 2015), whereas urban children in Ecuador exhibited lower HDL and higher LDL levels (Rosvik et al. 2022).

Although type 2 diabetes in childhood is still rare, the high prevalence of prediabetes among UEA schoolchildren should be interpreted as an epidemiological warning. Early detection of such alterations is crucial, especially given their potentially reversible nature through timely interventions in school and community settings (Sociedade Brasileira de Diabetes 2021).

These findings contribute to the global discourse on child health in underserved territories. Similar to many rural areas around the world, urban expansion zones lack adequate urban planning and access to preventive health services. By identifying metabolic vulnerabilities among schoolchildren living in these transitional spaces, the present study reinforces the importance of public health strategies tailored to the specific challenges of rural or rural‐like contexts, particularly in regions undergoing rapid urbanization.

While this study has certain limitations, they should be interpreted in light of its methodological strengths and its contribution to the understanding of health inequalities in childhood. First, the cross‐sectional design does not allow causal inferences between residential location and behavioral or clinical–metabolic outcomes. Moreover, the sample was drawn from a single municipality in southeastern Brazil, which may limit the generalizability of the findings to other regions with distinct socio‐spatial characteristics.

Behavioral data were collected using self‐reported questionnaires completed by caregivers and children, which may be subject to recall bias and social desirability—particularly regarding dietary intake and PA. Although validated instruments were employed (QUADA‐3 and QUAFDA), such limitations are inherent to studies involving pediatric populations.

Additionally, although several relevant clinical markers were evaluated, the lack of objective data on the environmental context (e.g., school distance, street quality, presence of public infrastructure) may restrict a broader understanding of structural barriers to PA and active commuting.

Finally, variables such as household income and parental education were self‐reported and presented a non‐negligible proportion of missing data, which may have introduced some degree of information bias or reduced the statistical power to detect certain associations.

Nevertheless, this study is among the first in Brazil to examine, in an integrated manner, how residential location influences metabolic markers, food consumption, and PA in schoolchildren. The findings provide important insights to inform territory‐sensitive public policies aimed at promoting child health and equity.

## 11 Conclusions

Adverse metabolic outcomes observed among schoolchildren in urban expansion areas can be explained by the rapid replacement of traditional foods with ultra‐processed products, the reduction in active commuting due to structural limitations, and lower paternal education, which reveals social vulnerabilities not captured by other socioeconomic indicators. As transitional territories, these areas simultaneously encompass the loss of protective behaviors from rural settings and the absence of consolidated urban resources, thus constituting critical zones for the emergence of health inequalities. Taken together, the results reinforce the need to recognize urban expansion areas as priority territories for integrated and place‐based public policies capable of preventing early metabolic disorders and reducing inequities in childhood.

## Data Availability

The data that support the findings of this study are available from the corresponding author upon reasonable request.
